# Emerging novel hydrogels application in oral and gastrointestinal diseases: design wet adhesive strategy towards possible mechanisms

**DOI:** 10.1080/14686996.2025.2556646

**Published:** 2025-09-11

**Authors:** Rongjun Xiao, Kai Huang, Yuwen Chen, Wentao Jiang, Laijun Xu

**Affiliations:** a Xiangya School of Stomatology, Central South University, Changsha, Hunan, China; b Department of Oral and Maxillofacial Surgery, Xiangya Hospital, Central South University, Changsha, Hunan, China; c School of Life Sciences, Central South University, Changsha, Hunan, China; d School of Stomatology, Shandong Second Medical University, Weifang, Shandong, China; e Stomatological Hospital, School of Stomatology, Southern Medical University, Guangzhou, China; f School of Stomatology, Changsha Medical University, Changsha, Hunan, China

**Keywords:** Wet adhesion, hydrogel, oral and gastrointestinal diseases, adhesive properties

## Abstract

The humid and highly dynamic milieu of the oral cavity and gastrointestinal tract poses formidable challenges to the precise localization and functionality of drugs and materials. Consequently, materials endowed with intrinsic wet adhesion properties hold great promise for the treatment of oral and gastrointestinal disorders. To a certain extent, the evolution of biomaterials has propelled progress in clinical diagnostics and therapeutic modalities. Wet-adhesive hydrogels, which can adapt to the moist and variable conditions of the digestive tract, display a spectrum of favorable biological attributes, such as antibacterial, anti-inflammatory, antioxidant, and hemostatic effects. These properties render them invaluable in the precision treatment of oral and gastrointestinal diseases. In this review, we scrutinize the adhesion mechanisms of wet-adhesive hydrogels and explore how their biological functions enable them to function efficiently within the gastrointestinal environment.

## IMPACT STATEMENT

This review introduces innovative wet-adhesive hydrogels designed for oral-gastrointestinal diseases, integrating wet-adhesive strategies and multifunctional properties to overcome environmental challenges and enhance treatment efficacy.

## GRAPHICAL ABSTRACT

**Figure:**
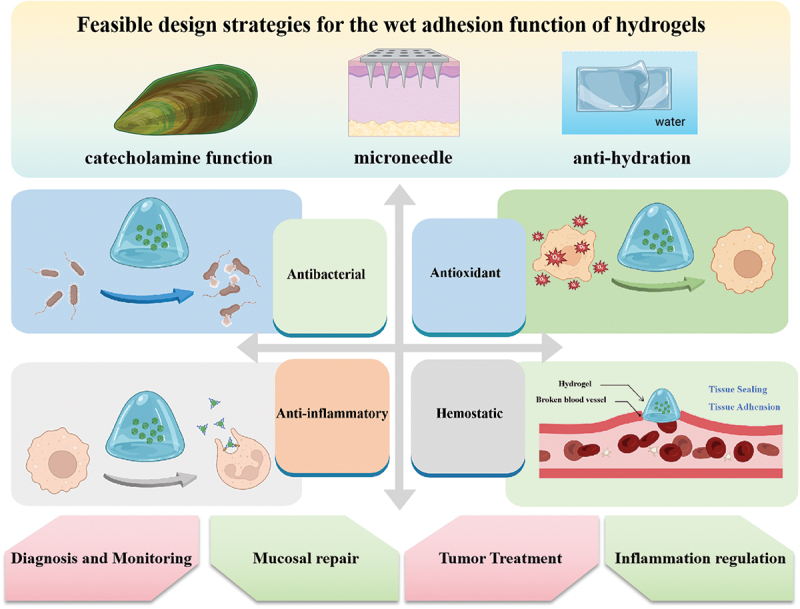

## 1. Introduction

The digestive tract, a continuous conduit extending from the mouth to the anus, is partitioned into the oral cavity, pharynx, esophagus, stomach, small intestine, and large intestine. Its principal functions encompass food digestion, nutrient absorption, and waste excretion [1]. Gastrointestinal diseases are common, cause considerable suffering, and can be fatal. GI diseases account for substantial healthcare use and expenditures [2]. Conditions such as gastric ulcers, colitis, and numerous oral diseases highlight the digestive system as an area with a high disease incidence. Furthermore, the complex milieu, characterized by diverse food residues and digestive fluids due to the physiological functions of the digestive system, imposes stringent demands on treatment modalities. Current drug delivery systems frequently struggle to adapt to the moist and highly dynamic environment of the oral and gastrointestinal tract. As a consequence, drugs may become inactivated or fail to reach the site of the lesion before they can exert their therapeutic effects [3].

Hydrogels are hydrophilic materials consisting of a three-dimensional polymer network. This network can be formed from synthetic polymers, natural polymers, or their derivatives. Hydrogels possess a remarkable capacity to absorb copious amounts of water [4,5]. Traditional hydrogels typically feature a simple structure, exhibit low mechanical strength, and lack specialized functions. These limitations significantly restrict their range of applications. However, hydrogels can be modified through appropriate techniques. Such modifications enable the fabrication of hydrogels with diverse structures, enhanced activities, and tailored functions [6]. Hydrogels have shown remarkable functions in several fields such as wound healing, cancer treatment, and locomotor system injury repair [7]. Hydrogels possessing adhesive properties can function in areas like wound sealing, drug delivery, and biosensors. However, achieving effective adhesion of hydrogels within a humid and highly dynamic milieu, such as the gastrointestinal tract, represents a formidable challenge that demands resolution [8]. Water molecules are regarded as an adverse obstacle to the robust adhesion of biomaterials. This is because water molecules can disrupt the adhesion of hydrogels to target tissues and organs, giving rise to feeble adhesion and, in the end, even leading to adhesion failure [9]. Consequently, there is an acute necessity to develop hydrogel materials featuring excellent wet-adhesion properties to further propel the application of hydrogels in biomedicine.

Recent reviews on wet-adhesion hydrogels predominantly address adhesion mechanisms and design principles [10–12]. Crucially, these features must align with specific application scenarios. Inspired by the humid, dynamic environments of the oral cavity and gastrointestinal tract, such hydrogels demonstrate significant potential for oral-gut applications. Despite shared anatomical and physiological continuity throughout these regions, existing literature overlooks hydrogel applications across this integrated system. Therefore, integrating recent advances in wet-adhesive hydrogels, we analyze their evolution from adhesion mechanisms through derived properties to diverse biomedical applications for treating oral-gut disorders.

This review paper comprehensively examines the most recent research advancements regarding wet-adhesion hydrogels. It meticulously elaborates on the underlying mechanism of wet adhesion, and systematically summarizes the biological properties of wet-adhesion hydrogels as well as their applications in the prevention and treatment within the realm of oral and gastrointestinal diseases. Additionally, it is put forward that in future research endeavors, efforts should be made to mimic the intricate microenvironment of the digestive tract to the greatest extent possible. Moreover, it is crucial to rationally coordinate and plan diverse biological events, thereby facilitating the translation from scientific research findings to clinical practice.

## 2. Feasible design strategies for the wet adhesion function of hydrogels

**Figure 1.**
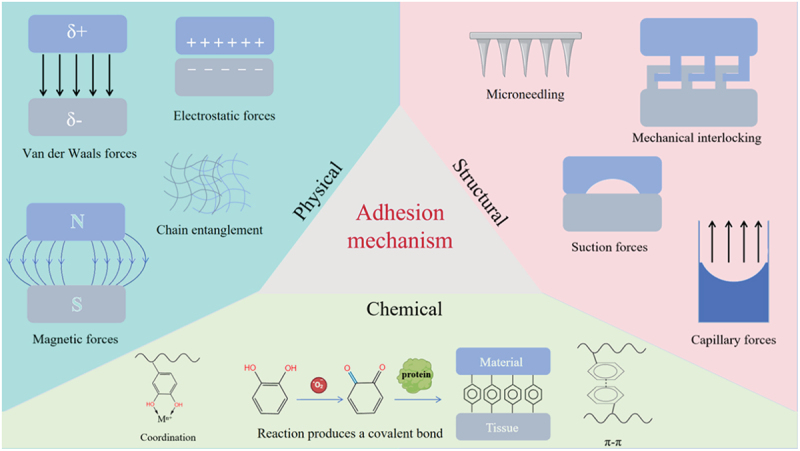
Adhesion mechanism of hydrogels.

In principle, the phenomenon of adhesion can be succinctly defined: when the energy required to separate two dissimilar objects exceeds that needed to establish contact, the objects will adhere to one another. However, when these objects are more complex than a pair of well-defined molecules situated within a controlled environment, where attractive forces surpass repulsive interactions, adhesion transforms into a multifaceted process. This intricate phenomenon encompasses a variety of mechanisms that operate across diverse spatial scales [13]. Wet adhesion is a prevalent phenomenon in nature, particularly evident in hydrogels, where the adhesion process is governed by three key factors: physical, chemical, and structural (Figure 1) [14]. Building on the aforementioned adhesion factors, effective strategies for underwater or wet adhesion involve engineering these factors into hydrogels in such a way that they operate complementarily, additively, or even multiplicatively. In other words, the adhesion factors must enhance one another’s functionalities, address individual shortcomings, and mitigate potential failures [15]. Among current design strategies for achieving wet adhesion in hydrogels, the most prevalent approaches leverage catecholamine functionalization, microneedle structures, and hydrophobic surface modifications. This section will systematically introduce these three established mechanisms. Additionally, we will discuss emerging bioinspired strategies derived from natural organisms that offer alternative pathways for robust wet adhesion.

### 2.1. Wet adhesive based on catecholamine function

**Figure 2.**
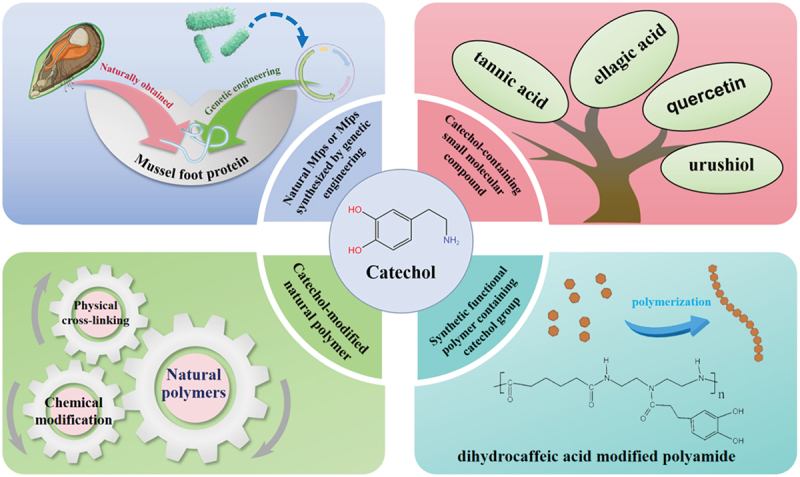
Catechol derivatives applied to wet tissue adhesive.

Researchers have derived inspiration from marine organisms, with mussels being a notable example. They have extracted pivotal chemical groups from mussel foot proteins (Mfps), which are instrumental in the adhesion process [8,16]. In current design strategies aimed at endowing hydrogels with wet-adhesion capabilities, the catecholamine-containing compounds can be categorized into the following four types, as shown in Figure 2. Among these compounds, dihydroxyphenylalanine (DOPA) has drawn remarkable attention. It has inspired researchers to integrate DOPA into a wide array of hydrogel molecular frameworks. This integration has substantially augmented the diversity of artificial wet-adhesion hydrogels, thereby enabling their successful applications in areas such as tissue sealing, drug delivery, and physiological signal detection [9,17,18]. The catechol moieties within catecholamines are capable of manifesting transient adhesion. This occurs via non-covalent interactions between the wet-adhesive material and the targeted substrate. Such interactions encompass hydrogen bonds, metal-ion coordination bonds, cation-π bonds, and extended π-bonds [19]. Researchers have employed catecholamines to chemically modify pre-existing materials with the aim of enhancing the wet adhesion of hydrogels. For example, Xujie Zhou and colleagues modified chitosan (CS) using catechol groups. This modification led to an improvement in both its mucoadhesive and hemostatic properties [20]. Yanhua Zhao and his/her team developed a kind of smart material that features switchable adhesive characteristics in wet environments. This adhesive is mainly composed of two copolymers: a mussel-mimicking guest-adhesive copolymer and a thermoresponsive host copolymer [21]. In the natural world, the adhesive proteins secreted by mussels possess catechol groups. These groups can readily oxidize into quinones, leading to a forfeiture of adhesive properties when the pH value surpasses 5.5. To address this issue, mussels acidify the surface beneath their feet to around pH 2. By doing so, they establish a reducing environment that safeguards the adhesive characteristics of their proteins during the deposition process [22–24]. Consequently, the environment in which catecholamine-based hydrogel materials are placed is of paramount importance in dictating the strength of their wet adhesion. In the realm of dental adhesives, phosphoric-acid-based etching is employed for bonding to dentin. Owing to the similarities between dental adhesives and underwater mussel adhesives, Dohoon Lee and colleagues extended and assessed dentin and zirconia dental adhesives. They achieved this by integrating mussel-inspired (DOPA) – thiol redox chemistry with acid etching, thereby enhancing the adhesives’ adaptability to wet working conditions [25]. Wet tissue adhesives featuring catechol groups show great potential in a wide range of fields, such as tissue repair, drug and cell delivery, and flexible electronic materials. However, for large-scale practical implementation, several key challenges need to be overcome. These include strengthening mechanical strength, guaranteeing long-term stability under ambient conditions, controlling material costs, and optimizing biosafety and biodegradability. Additionally, although many catecholamine-based hydrogels possess robust and adaptable interfacial bonding abilities, their failure to enable non-destructive and intelligent detachment of biofunctional devices from living organisms underscores the urgent requirement for progress in reversible adhesion technologies.

### 2.2. Microneedle-based wet adhesive mechanism

**Figure 3.**
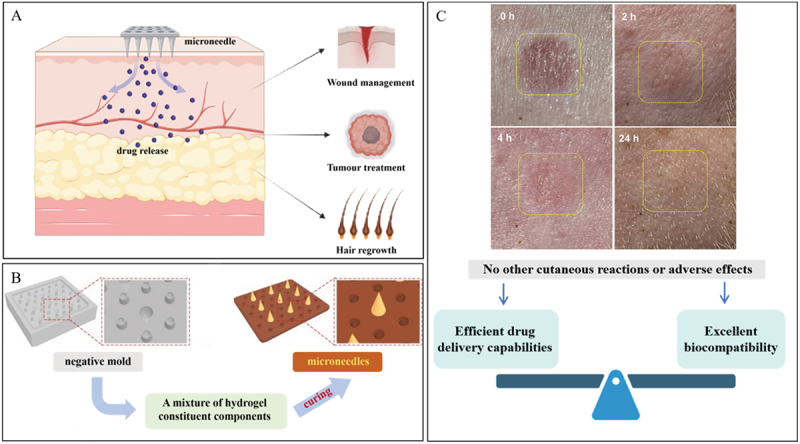
The design concept and unique advantages of microneedles. A. Application of hydrogel microneedle device. B. Fabrication and characterization of the bioinspired multifunctional microneedles. Reproduced by permission from [27], copyright 2020, AAAS. C. Skin irritation after application of liraglutide MN patches to minipigs. Reproduced by permission from [28], copyright 2024, Springer.

A microneedle device is composed of micron-sized needles that are arrayed on a small patch. The distinctive features of this technology include a more rapid onset of action, enhanced patient compliance, the convenience of self-administration, as well as improved permeability and therapeutic efficacy [26]. Hydrogel microneedles achieve effective underwater adhesion through the synergistic interplay of intermolecular interactions and mechanical interlocking. Hydrogel microneedles have exhibited outstanding performance in the treatment of diverse diseases (Figure 3A). Drawing inspiration from the adhesion mechanisms of octopus tentacles, researchers have engineered microneedles equipped with multifunctional adhesives. Xiaoxuan Zhang and his/her team have reported the development of desirable multifunctional microneedles designed for versatile transdermal drug delivery [27]. Zhou Zhu and his/her colleagues developed bionic suction cups that enable microneedles to remain firmly in position even in wet conditions. They achieved promising therapeutic effects, such as accelerating the healing process of ulcers and arresting the early progression of tumors [29].

These multifunctional microneedles were fabricated through the replication of a specifically designed negative mold. As illustrated in Figure 3B, they feature conical tips that are orderly aligned, along with the surrounding concave chambers. Moreover, these conical tips possess sufficient mechanical resilience, allowing them to penetrate soft tissues without the risk of breakage. The microneedles can effectively interlock with the tissue while causing minimal physical damage to themselves [30]. Daehoon Han and his associates designed a microneedle featuring bioinspired backward-facing curved barbs. Their experimental findings demonstrate that the tissue adhesion capacity of a backward-facing barbed microneedle is 18 times greater than that of a barbless microneedle [31]. Yan-Wen Ding and his/her team designed a hyaluronic acid (HA)-based hydrogel-formed microneedle, which offers a safe and effective treatment option for androgenetic alopecia. The combination of the HA-based hydrogel with polyhydroxyalkanoates nanoparticles remarkably improved the mechanical properties of the microneedles and increased the efficiency of skin penetration [32]. Microneedles are outstanding drug delivery systems that exhibit excellent wet adhesion and typically possess favorable biocompatibility. Due to their minimally invasive nature, they effectively minimize the occurrence of side effects such as inflammation (Figure 3C) [28]. In order to boost the wet adhesion of hydrogel materials by means of the microneedle structure, it is essential to take into account the mechanical strength of the hydrogel materials. Additionally, the shape, arrangement pattern, and the design of the negative mold for the microneedles are all of vital significance.

### 2.3. Wet adhesive mechanisms based on hydrophobic modification

**Figure 4.**
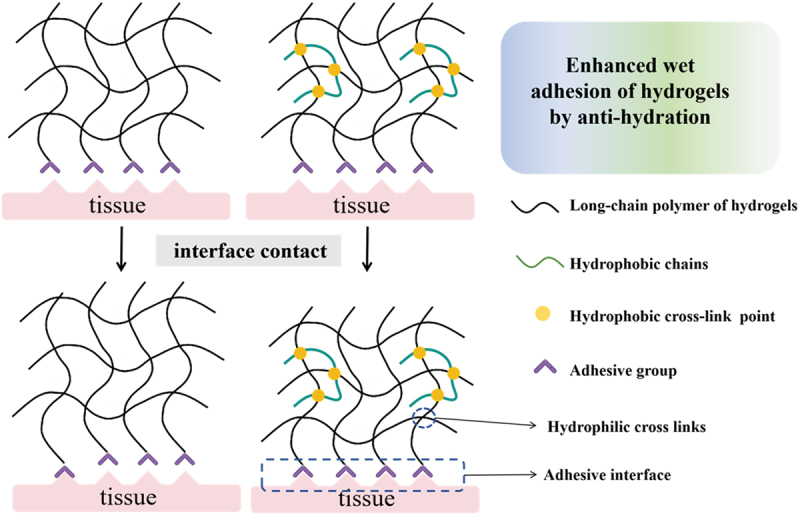
Enhanced wet adhesion of hydrogels by anti-hydration.

At the microscopic level, the total interfacial adhesion properties of tissue adhesives are actually the sum of adhesion and cohesion [33,34]. However, it is an undeniable fact that when hydrogel adhesives are in operation at the wet tissue interface for an extended period, water molecules infiltrate the network of the adherent materials. This infiltration disrupts the interfacial adhesion and the intrinsic strength of the adhesive, consequently restricting its effectiveness in wet adhesion [35]. When an adhesive is immersed in an aqueous milieu, hydrophobic molecules can efficiently disrupt the hydration process. This property not only facilitates the maintenance of the material’s cohesion but also significantly enhances the interface’s drainage capacity [36,37]. Consequently, the hydrophobic modification of adhesives represents a crucial dimension in augmenting their wet-adhesion capabilities (Figure 4).

Yue Hou and colleagues explored the impact of varying hydrophobic chain lengths on the wet adhesion of hydrogels [38]. In their study, the primary hydrophilic network consisted of either polyacrylamide (PAM) or poly(acrylic acid) (PAA), in combination with a series of hydrophobic monomers of different chain lengths. Notably, when ethyl acrylate was employed as the hydrophobic monomer, it exhibited the optimal wet-adhesion effect. Long hydrophobic chains tend to form extensive hydrophobic entanglements within the polymer network. This, in turn, results in excessive cohesion within the hydrogel network. As a consequence, during the detachment of the hydrogel from substrate surfaces, the hydrogel becomes rigid [14,39]. Therefore, external pulling forces can readily break the adhesive bonds between the hydrogel and the substrate. Heng An and co-workers introduced hydrophobic chains as cross-linking points via a simple and versatile method. This approach enabled the preparation of hydrogels that possess anti-hydration properties, toughness, and high wet-state adhesion [40]. Gelatin – polyacrylic – ethylene dimethacrylate (GAE) hydrogels were synthesized by adding ethylene dimethacrylate (EDMA) for polymerization on the basis of gelatin – polyacrylic (GA) hydrogels. In this work, a novel strategy was proposed: cross-linking a small quantity of hydrophobic chains with the hydrophilic chains of cross-linkers. This strategy not only enhanced the wet adhesion of the hydrogels but also maintained the hydrogels’ constant size and stability. Xinyue Wang et al. prepared mTA-PAA/PEI PECA hydrogels by mixing modified tannic acid (mTA, an electrostatic complex of tannin and hydrophobic long chains) with anionic acrylic acid (AA) and the cationic polymer PEI [41]. This hydrogel is proved that it has tough mechanical strength and adhesion strength to wet tissue. Chao Fu et al. fabricated a hydrogel capable of robust adhesion in both aqueous and oil environments. This hydrogel was mainly synthesized through the copolymerization of acrylamide (AAm) and octadecyl methacrylate (OMA) [42]. It demonstrated non-specific adhesion on diverse substrates in a wide range of aqueous solutions, including water, high-salt aqueous solutions, simulated body fluids, acidic aqueous solutions, and basic aqueous solutions. Moreover, it adhered well in organic reagents such as ethanol, DMSO, silicone oil, toluene, and dichloromethane, as well as in oils like cyclohexane, dodecane, and castor oil. Guang Huang et al. successfully synthesized hybrid hydrophobic hydrogels that exhibit strong adhesion to a variety of substrates [43]. These hybrid hydrogels, which contain both hydrophobic groups (ethyl acrylate, EA) and ionizable groups (cationic macro-ions, DAC), were prepared through free-radical copolymerization in a dimethyl sulfoxide (DMSO) solution. As a result, the enhanced adhesion can be ascribed to the synergy between two types of interactions: hydrophobic associations and dynamic Coulombic interactions. Bo Yi et al. proposed a simple yet versatile strategy involving the induction of surface network reconfiguration to regulate the surface wettability and bio-adhesion of hydrogels without changing their bulk chemical and physical properties. Different hydrogels based on various polymers, such as PMAA (poly-methacrylic acid), PAA, and PAAm (polyacrylamide), showed a significant enhancement in wet adhesion following the hydrophobic modification of their surfaces [33]. The synergistic action between hydrophilic and hydrophobic polymer chains was accountable for eliminating the interfacial liquid layer and enabling effective interfacial bonding during wet adhesion. When devising the hydrophobic modification of hydrogels, it is essential to take into account the impacts of hydrophobic groups on the swelling, mechanical strength, and biocompatibility of hydrogels.

In conclusion, hydrophobic modification is of pivotal importance in augmenting the wet adhesion of hydrogels, especially in dynamic and moisture-laden environments like the gastrointestinal tract. Employing diverse strategies, such as the introduction of hydrophobic chains or the modification of hydrogel surface properties, researchers have successfully demonstrated enhanced adhesion strength and stability (Table 1). The incorporation of hydrophobic interactions not only fortifies the cohesion within the hydrogel network but also mitigates hydration effects, thus improving the material’s performance under wet conditions. These progressions suggest that the meticulous design of hydrophobic-modified hydrogels holds great promise for a broad spectrum of applications, spanning from tissue adhesives to biomedical devices.

**Table 1.** Structural composition of hydrophobically modified hydrogels.

hydrophilic network	Hydrophobic groups	Main Characteristics	Reference
PAM or PAA	methyl acrylate, ethyl acrylate, butyl acrylate, hexyl acrylate, dodecyl acrylate	only the monomers with proper short chain length give rise to the strongest wet adhesion	Yue Hou et al. [38].
GA	EDMA	maintains the hydrogel constant size of the gel as well as its stability.	Heng An et al. [40].
AA and PEI	mTA	tough mechanical strength	Xinyue Wang et al. [41].
AAm	OMA	nonspecific adhesion on various substrates in various aqueous solutions	Chao Fu et al. [42].
DAC	EA	the hydrophobic associations and the dynamic Coulombic interaction	Guang Huang et al. [43]
PAA, PMAA, PAAm	silicone chains	maintain hydrogels’s bulk chemical and physical properties	Bo Yi et al. [33]
poly acrylic acid and chitosan	alkyl chain	the hydrophobic moiety shields catechol from oxidation into a less adhesive quinone	Xianmou Fan et al. [44].
polyethylene imine	thioctic acid	Thioctic acid results in an in-situ conversion of the condensate-hydrogel without any additional stimulation	Xin Peng et al. [45].
Poly vinyl alcohol and PAA	tannic acid	TA has antibacterial and hemostatic functions while exerting hydrophobic effects	Jae Park et al. [46].

### 2.4. Biomimetic hydrogel designs with wet adhesion function

Besides the three aforementioned wet-adhesion modification strategies, researchers have also developed several novel chemical modification strategies for material surfaces, drawing inspiration from natural organisms. These strategies aim to endow materials with more efficient wet-adhesion capabilities.

**Figure 5.**
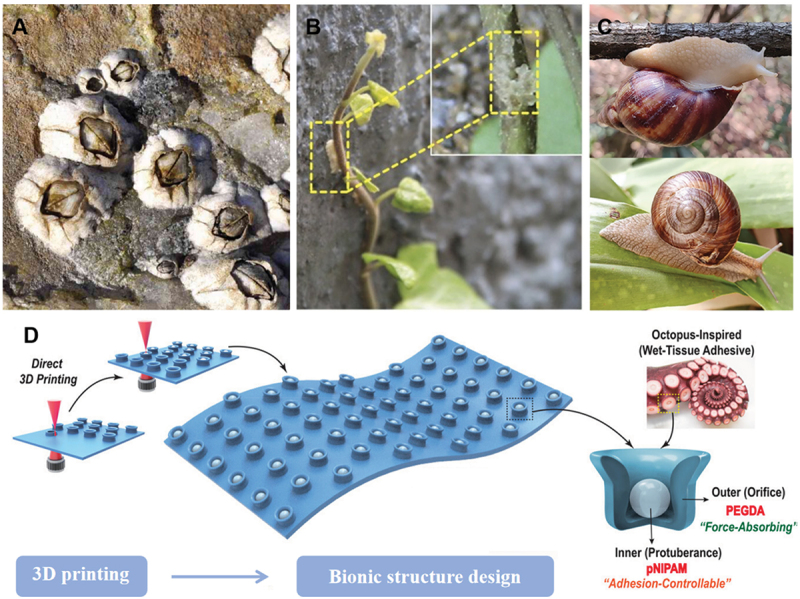
Wet adhesion in nature. A. Barnacles stick to the rocks. B. Ivy shoots attached to the wall. Reproduced by permission from [47], copyright 2016, PANS. C. Snails stick to plants. Reproduced by permission from [48], copyright 2023, nature portfolio. D. Octopus-inspired 3D printable wet adhesive hydrogel. Reproduced by permission from [49], copyright 2022, Wiley.

Barnacles employ a system of amyloids to establish stable waterborne adhesion on solid surfaces (Figure 5A) [50,51]. Inspired by this phenomenon, Rongrong Qin et al. modified the surface of wet materials with protein nanomembranes. During amyloid aggregation at the air/water interface, the exposed amino acid residues can substitute the hydrogel surface. This substitution facilitates the formation of adhesion between the protein nanomembrane and the wet surface [52]. The adventitious roots of English ivy (Hedera helix) secrete a yellowish mucilage (Figure 5B). This viscous exudate is abundant in spherical nanoparticles, which enhance the plant’s ability to climb vertical surfaces [47,53]. As the mucus evaporates, the globular nanoparticles within it become concentrated and form a permeable membrane. This membrane allows for mechanical interlocking between the root and the substrate surface. Tuo Deng et al. develop a natural biological adhesive from snail mucus gel, which consists a network of positively charged protein and polyanionic glycosaminoglycan. The malleable bulk adhesive matrix can adhere to wet tissue through multiple interactions (Figure 5C) [48]. Yun-Woo Lee et al. used 3D printing technology to simulate the tentacle structure of an octopus using hydrogel to enhance wet adhesion (Figure 5D) [49]. The elaborate microstructure and chemical constitution of solid-material surfaces play a decisive role in determining the performance of interfacial adhesion. When designing wet-adhesion hydrogels, a variety of surface interactions merit attention. These include surface and field forces (such as van der Waals forces, electrostatic and magnetic forces), material-bridging mechanisms (for instance, capillary forces, diffusion, and mechanical interlocking), liquid surface tension, suction, and chemical bonding [54]. In summary, surface-modification techniques are crucial for augmenting the wet-adhesion properties of materials. The inspiration drawn from nature has enabled the discovery of diverse and highly effective adhesion mechanisms. From the amyloid-based systems of barnacles to the mucilage excretions of English ivy and the mucus gel of snails, these natural adhesives provide invaluable insights into how surface interactions and chemical modifications can be exploited to enhance adhesion to wet surfaces. By emulating these biological systems and integrating advanced technologies like 3D printing, researchers can engineer hydrogels with complex microstructures and customized surface chemistries to achieve exceptional wet-adhesion performance. The combination of multiple surface interactions, including van der Waals forces, electrostatic interactions, capillary forces, and chemical bonding, further elevates the performance of wet-adhesive hydrogels.

## 3. Advanced functions of wet adhesion hydrogels

**Figure 6.**
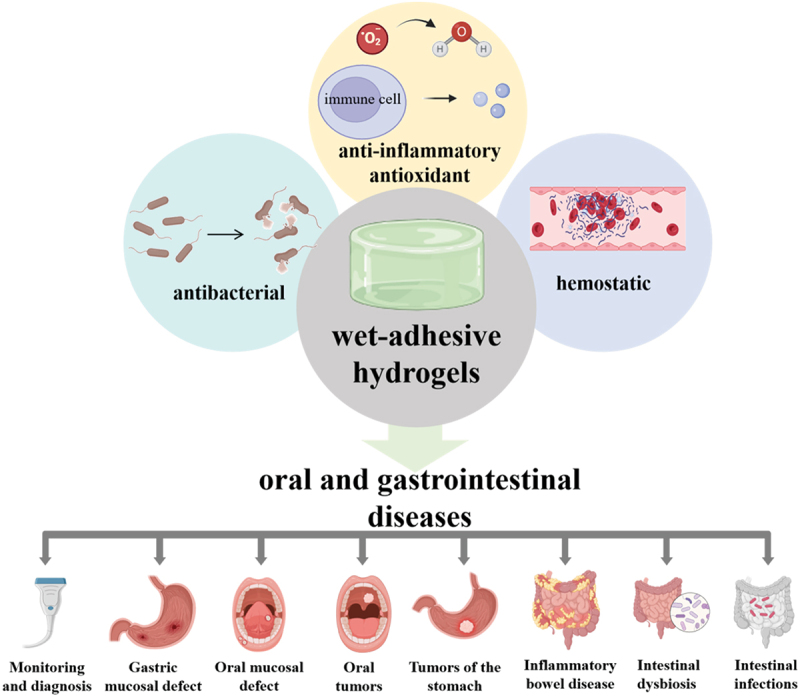
Applications and functions of wet adhesive hydrogels in oral and gastrointestinal diseases.

The physiological functioning of the digestive system dictates that it is a complex lumen filled with food debris and digestive juices. This moist and highly dynamic milieu imposes exacting requirements on the treatment of digestive tract diseases [7]. Wet-adhesion hydrogels demonstrate remarkable adaptability to the unique environments of the oral cavity and gastrointestinal tract. As basic research delves deeper, an array of hydrogel dressings featuring enhanced single or multiple biological functions has started to materialize (Figure 6). In this part, we expound on the recent progress in wet-adhesion hydrogels from the vantage point of functional modification [10,55,56].

### 3.1. Antibacterial properties of wet adhesion hydrogel

**Figure 7.**
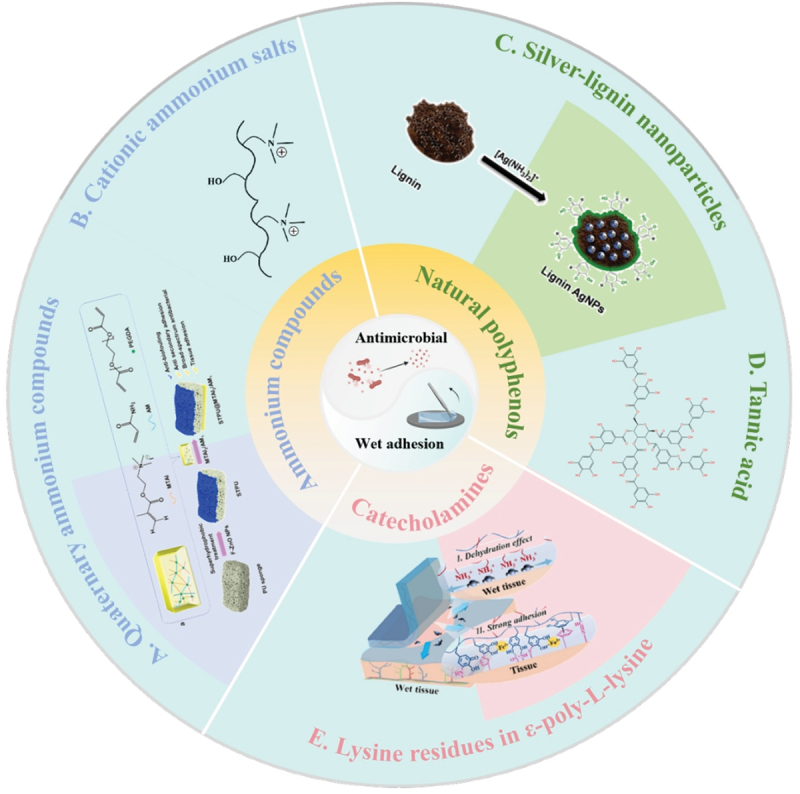
The chemical composition that gives the hydrogel its antibacterial and wet adhesion ability. A. Quaternary ammonium compounds. Reproduced by permission from [63], copyright 2024, Elsevier. B. Cationic ammonium salts. C. Silver-lignin nanoparticles. Reproduced by permission from [64], copyright 2020, American chemical Society. D. Tannic acid. E. Lysine residues in ε-poly-L-lysine. Reproduced by permission from [65], copyright 2023, Elsevier.

A minute quantity of bacteria that adhere to the wound surface can proliferate rapidly, culminating in the formation of a dense bacterial biofilm, which ultimately leads to infection [57]. Diseases of the digestive system are particularly vulnerable to infection by a diverse range of pathogenic bacteria. For example, Helicobacter pylori is the primary causative agent of gastric ulcer and gastritis and is also associated with an elevated risk of gastric cancer. Additionally, Escherichia coli, Salmonella, Shigella, Streptococcus, among others, can also cause infections in the digestive system [58]. These bacterial infections can give rise to diverse digestive-system diseases. Gastrointestinal infections, food poisoning, and gastric ulcers are among the common manifestations. In more severe scenarios, they can trigger serious complications like intestinal perforation or infectious enteritis. Notably, when bacterial adhesion takes place prior to tissue repair, the host immune system becomes hampered in its ability to specifically target the wound surface [59]. Bacterial infection is often an unavoidable issue during wound repair. Moreover, bacterial infection can disrupt the structure of adhesives, diminishing their adhesive strength and thus restricting their application [60]. Wet-adhesive hydrogels equipped with antimicrobial capabilities evidently hold great promise for applications in the treatment of the digestive tract [61,62]. The antimicrobial elements in wet-adhesion hydrogels can be divided into three main categories: ammonium compounds, polyphenols, and lysine-containing compounds (Figure 7A–E). Siyu Li and colleagues designed a reversibly adhesive hydrogel through the free-radical copolymerization of the cationic monomer [2-(acryloyloxy)ethyl]trimethylammonium chloride, the hydrophobic monomer ethylene glycol phenyl ether acrylate (PEA), and N-isopropyl acrylamide [66]. This hydrogel not only exhibits anti-swelling and antimicrobial characteristics but can also be readily detached as required. Hang Xu et al. reported a multilayered wound dressing (STPU@MTAI2/AM1), the hydrogel attached to the inner layer of the material acts as a binder and antimicrobial agent (Figure 7A) [63,67,68]. Yicheng Lv et al. designed a multifunctional hydrogel (OD/EPL@Fe) composed of catechol-modified oxidized hyaluronic acid (OD), ε-poly-L-lysine (EPL), and Fe3. Lysine residues in hydrogels play a key role in underwater adhesion, which can replace hydrated cations on wet surfaces, allowing catechol groups to bind tightly to tissues, thus providing high wet adhesion (Figure 7B,E) [65]. Samson Afewerki et al. designed a lignin-based multifunctional antimicrobial hydrogel, the hydrogel has strong antimicrobial activity and wet adhesion (Figure 7C) [64]. Lignin, as a sustainable material, is capable of serving as both a reducing agent and a stabilizer for the preparation of various metal-based nanoparticles (NPs). Through this dual role, it enables the generation of NPs and simultaneously activates the catechol functionality inherent in lignin [69]. In Jae Park et al’.s research, tannic acid (TA) is introduced into a double network hydrogel consisting of poly(vinyl alcohol) and poly(acrylic acid) to realize a tough, self‐healable, nonswellable, conformally tissue‐adhesive, hemostatic, and antibacterial hydrogel [70]. The addition of tannic acid not only imparts wet adhesion and antibacterial properties to the hydrogel but also improves the physical and chemical properties of the hydrogel (Figure 7D) [71]. The multilayered wound dressing has demonstrated the ability to promote wound healing when addressing Methicillin-resistance Staphylococcus aureus (MRSA)-infected wounds. In summary, the antimicrobial attributes of wet-adhesive hydrogels are of paramount importance for their utilization in the treatment of digestive system disorders. These developments underscore the substantial promise of wet-adhesive hydrogels as adaptable and efficacious therapeutic agents for combating infections and facilitating tissue repair within the gastrointestinal tract.

### 3.2. Antioxidant and anti-inflammatory properties of wet adhesion hydrogels

**Figure 8.**
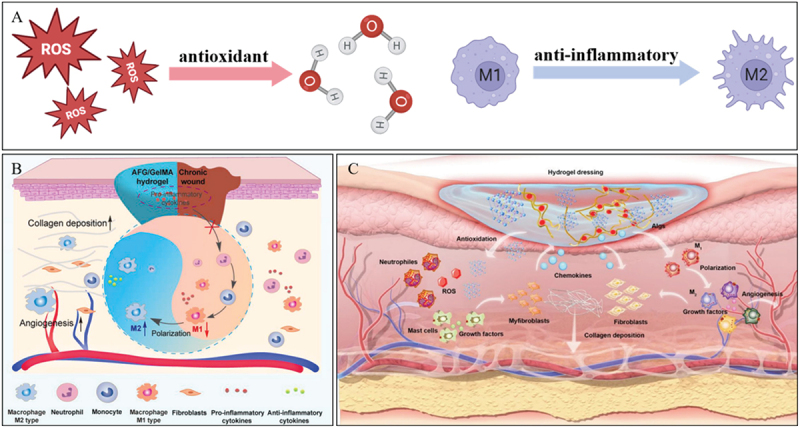
Mechanism of action of hydrogel anti-inflammatory and antioxidant properties. A. Schematic diagram of antioxidant and anti-inflammatory principles. B. Activity and mechanism of hydrogel anti-inflammatory properties promoting chronic wound healing. Reproduced by permission from [75], copyright 2023, Elsevier. C. Tunable sulfated alginate-based hydrogel with enhanced anti-inflammatory and antioxidant capacity for promoting wound repair. Reproduced by permission from [76], copyright 2023, BMC.

Maintaining the equilibrium of intracellular redox states, namely, achieving effective antioxidant effects, holds the promise of averting abnormal cell proliferation and immune response dysfunctions. As anticipated, it has been substantiated that antioxidation can expedite wound healing processes and sustain normal immune functions [72–74]. Numerous investigations have been centered around the exploration and enhancement of the antioxidant characteristics of wet-adhesion hydrogels, as well as the examination of their pivotal roles in the therapeutic management of various diseases (Figure 8A) [77,78].

Liucan Wang et al. proposed a catechol chemistry-mediated core-shell nanoplatform aimed at scavenging reactive oxygen species (ROS) and alleviating oxidative stress [79]. Polydopamine nanoparticles possess the ability to function as an electron reservoir, effectively quenching highly oxidized and toxic free radicals. Quercetin (QT), a flavonoid compound commonly present in fruits and vegetables, imparts excellent antioxidant properties to the hydrogel [80]. Leveraging dopamine and QT, a series of multifunctional hydrogel wound dressings were ingeniously designed and fabricated. These dressings exhibit remarkable properties, including high adhesiveness, self-healing capacity, antioxidant activity, and antibacterial performance [81]. Changkai Yang et al. engineered a hydrogel formulation that incorporated collagen and hyaluronic acid [82]. The presence of polyphenolic compounds, specifically catechol gallic acid within the hydrogel matrix, endows it with both superior wet adhesion capabilities and notable antioxidant properties [83,84]. Prussian blue nanoparticles (PBNPs) function as antioxidant nanozymes, thereby enabling rapid and efficient scavenging of ROS [85,86]. Can Huang et al. constructed an innovative anti-inflammatory hydrogel platform. This platform is based on tunable sulfated alginates (Algs) that exhibit remarkable chemokine sequestration performance, as well as on Prussian blue (PB) nanozymes which are well-known for their outstanding antioxidant activity (Figure 8C) [76].

Inflammation represents an intrinsic defense mechanism orchestrated by immune cells and cytokines. Its primary purpose is to facilitate the repair of damaged tissues and eliminate a diverse array of harmful agents, including pathogens. Indeed, any element capable of inflicting damage to cells or tissues has the potential to trigger an inflammatory response [87]. However, when inflammation becomes excessive, and is accompanied by the generation of a substantial quantity of reactive oxygen species (ROS), such as hydroxyl radicals, hydrogen peroxide, superoxide anions, and nitric oxide, among others, it frequently causes cell damage. This occurs through the triggering of deoxyribonucleic acid (DNA) oxidation, protein oxidation, and lipid peroxidation processes. Consequently, it gives rise to numerous pathological dysfunctions within the body. In what follows, we will explore wet adhesion hydrogel-based anti-inflammatory therapeutic approaches, encompassing strategies related to ROS scavenging, the blockage of inflammatory pathways, and drug delivery mechanisms [88,89].

Immune responses are regulated by signals emanating from the tissue microenvironment. Besides biochemical signals, mechanical cues and forces generated by the tissue itself, its extracellular matrix, and the cells that make it up also influence and mold the function of immune cells [90,91]. Vijaykumar S. Meli and his colleagues have reported that the adhesive microenvironment has the ability to modulate the inflammatory response of macrophages via the transcriptional coactivator YAP [92]. They discovered that when macrophages adhere to soft hydrogels, it leads to a reduction in inflammation as opposed to when they adhere to stiff materials. This phenomenon is correlated with decreased expression of YAP and its reduced localization within the nucleus. Furthermore, Xu et al. put forward the concept of a chitosan-catechol mucoadhesive gel. This gel has the advantage of enabling the more effective and safer delivery of sulfasalazine compared to oral administration. As a result, it can assume a crucial role in the therapeutic management of ulcerative colitis [93]. Zhipeng Zhou and his team developed a double-network hydrogel biomaterial. This biomaterial is composed of snail glycosaminoglycan (AFG) and methacrylated gelatin (GelMA), with AFG being the key bioactive ingredient derived from snail mucus (Figure 8B) [75]. The hydrogel demonstrated a remarkable ability to reduce inflammation. It achieved this by sequestering pro-inflammatory cytokines. Additionally, it downregulated the expression of these cytokines through the inhibition of the NF-κB signaling pathway. Moreover, the hydrogel was also capable of promoting the polarization of macrophages towards the M2 phenotype. Xinyue Ge et al. reported on a multifunctional hydrogel that is designed for the effective treatment of ulcerative colitis (UC). Once implanted in situ, this hydrogel can adhere securely to the ulcerative lesion. It has the capability to continuously release dexamethasone (DEX). Through the inhibition of the toll-like receptor 4 (TLR4)-nuclear factor kappa-light-chain-enhancer of activated B cells (NF-κB) axis, the released DEX can induce the repolarization of mucosal macrophages from the M1 phenotype to the M2 phenotype [94]. Recent breakthroughs in wet-adhesive hydrogels have unveiled substantial potential for alleviating oxidative stress and inflammation. Antioxidant-functionalized hydrogels, which incorporate compounds such as catechol, polyphenols, and Prussian blue nanoparticles, efficiently scavenge reactive oxygen species (ROS), thereby mitigating oxidative damage. For example, hydrogels laden with polydopamine nanoparticles or quercetin display augmented ROS-quenching and antioxidant characteristics. Meanwhile, those based on collagen and hyaluronic acid exhibit both antioxidant and adhesive prowess. Inflammation, frequently exacerbated by ROS, can be regulated via these hydrogels through cytokine scavenging, inhibition of pro-inflammatory signaling pathways such as NF-κB, and promotion of macrophage polarization towards the anti-inflammatory M2 phenotype. These multifunctional hydrogels present promising strategies for treating oxidative-stress-related disorders and enhancing tissue repair, particularly within the challenging milieu of the gastrointestinal tract.

The antioxidant mechanism of wet-adhesion hydrogels is mainly divided into three categories: free radical scavenging type, metal ion chelation type, and enzyme-mimicking antioxidant type (Table 2). We need to adjust the design strategy of wet-adhesion hydrogels according to the application environment, and the commonly used typical strategies include: (1) antioxidant-anti-inflammatory synergy (hydrogels inhibit the NF-κB pathway by clearing ROS and reduce the secretion of pro-inflammatory factors); (2) Adhesion-antioxidant synergy (dopamine groups provide wet adhesion and free radical scavenging ability synchronously); (3) Nanoenzyme-drug delivery synergy (nanoenzymes catalyze ROS decomposition and promote drug release).

**Table 2.** Classification of the mechanism of action of antioxidants in hydrogels.

Type	Mechanism	Example
Free radical scavenging	Directly captures ROS and interrupts the oxidative chain reaction	Dopamine/polydopamine: Provides electron neutralization of free radicals through phenol hydroxyl groups [79]
Metal ion chelating	Complexing transition metal ions， prevent them from catalyzing ROS generation	Quercetin (QT), containing polyphenol hydroxyl groups that chelate metal ions [80]
Enzymes mimic antioxidants	Mimics natural antioxidant enzymes catalyze the breakdown of ROS	Prussian blue nanoparticles (PBNPs) catalyze the decomposition of H_2_O_2_ to H_2_O [76]

### 3.3. Hemostatic properties of wet adhesion hydrogel

**Figure 9.**
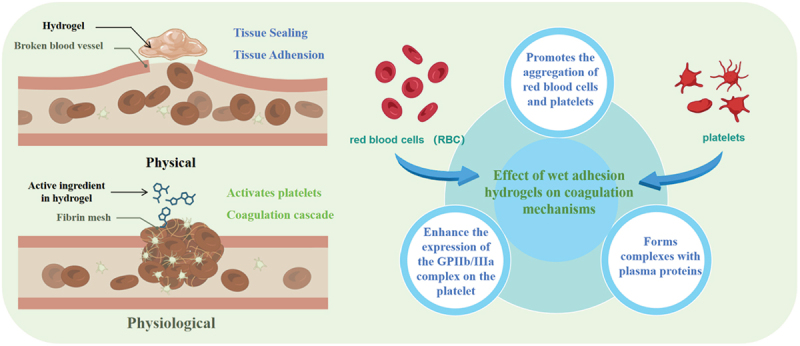
Wet adhesion hydrogel hemostatic function.

Gastrointestinal diseases frequently manifest bleeding symptoms during their progression. For instance, upper gastrointestinal bleeding represents a prevalent clinical emergency. As such, achieving effective and prompt hemostasis is of utmost importance in surgical procedures [95]. During gastrointestinal bleeding, it is challenging to pinpoint the bleeding site. Consequently, the development of hemostatic materials that are safe, reliable, and exhibit excellent hemostatic efficacy has emerged as a research focal point [96,97]. Owing to their biocompatibility, the ability to tune mechanical properties, and drug-loading capacity, certain hydrogels have been engineered for the repair of gastrointestinal perforations and have shown remarkable potential for clinical implementation. In recent times, wet-adhesion hydrogel composites have emerged as a novel class of hemostatic agents in disease treatment. These composites can augment hemostasis either by establishing physical conditions favorable to hemostasis or by directly intervening in the physiological hemostatic processes (Figure 9) [98–101].

Wen Yang et al. engineered a potent wet-tissue adhesive constructed from collagen and starch materials (CoSt). The CoSt hydrogel exhibits superior hemostatic efficiency compared to fibrin glue. This superiority stems from the synergistic effects of its robust wound-sealing property, remarkable ability to arrest red blood cells, and the activation of the hemostatic barrier membrane [102]. Dongjie Zhang et al. designed a carboxymethyl chitosan/polyaldehyde dextran (CMCS/PD) hydrogel as the first-aid tissue adhesive for effective trauma emergency management [103]. The aldehyde groups in PD facilitate the adhesion and aggregation of red blood cells (RBCs), platelets, fibrin, and other blood coagulation factors through Schiff-base reactions. This action activates the coagulation cascade, leading to the rapid formation of blood clots. Additionally, the CMCS molecules, with their positively charged nature, expedite the aggregation of RBCs and coagulation factors through electrostatic interactions, further contributing to the formation of clots [104,105]. Junjin Zhu et al. engineered a low-swelling adhesive hydrogel composed of gelatin methacrylate (GelMA), nano-clay, and tannic acid (TA). This hydrogel exhibits rapid hemostatic properties and potent anti-inflammatory capacity [106]. Previous studies reported that TA could serve as an effective hemostatic material. It could bind with proteins in the blood, leading to protein precipitation for the subsequent process of coagulation activation [107,108]. Recent progress in wet-adhesive hydrogels has revealed their highly promising hemostatic potential for trauma management. For instance, a hydrogel based on collagen and starch (CoSt) showcases hemostatic efficiency superior to that of traditional fibrin glue. This superiority can be ascribed to its firm wound-sealing ability, effective red blood cell (RBC) arrest, and activation of the hemostatic barrier. In a similar vein, carboxymethyl chitosan/polyaldehyde dextran (CMCS/PD) hydrogels leverage aldehyde groups to promote the aggregation of RBCs and coagulation factors, thereby triggering the coagulation cascade for the swift formation of blood clots. Moreover, hydrogels integrating materials like gelatin methacrylate, nano-clay, and tannic acid (TA) display both rapid hemostatic and anti-inflammatory attributes. TA, in particular, facilitates clot formation by binding to blood proteins and initiating the coagulation process. These innovations highlight the substantial potential of wet-adhesive hydrogels as efficient hemostatic agents in emergency scenarios.

## 4. Application in the diagnosis and treatment of oral and gastrointestinal diseases

### 4.1. Wet adhesion hydrogels for monitoring and diagnosis of oral and gastrointestinal diseases

Real-time monitoring and prompt diagnosis are of great significance in the treatment of chronic gastrointestinal tract diseases [109]. Gastrointestinal (GI) residence systems have emerged as a highly promising field for the diagnosis and treatment of GI disorders. In contrast to conventional drug pills and implantation systems, ingestible hydrogel-based GI residence systems can be designed to have minimal invasiveness and multiple functions. As a result, they can effectively tackle problems associated with patient non-compliance, and also enable the monitoring and treatment of chronic diseases [110–113].

Hydrogen sulfide (H2S) is a representative metabolite produced by anaerobic pathogens during the degradation of salivary sulfur-containing amino acids, so salivary H2S has been considered as a potential biomarker for periodontitis monitoring [114]. Jingying Pan et al. propose a wearable radio frequency sensor for periodontitis monitoring based on an agarose hydrogel integrated split-ring resonator containing conjugated silver nanoparticles (AgNPs)-chlorhexidine (CHL) [115]. This hydrogel achieves the purpose of monitoring the development of periodontitis by monitoring the content of H2S in periodontitis lesions.

**Figure 10.**
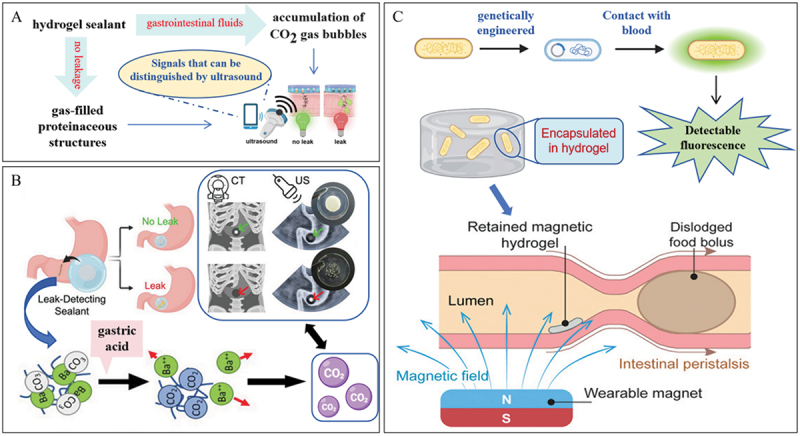
Wet adhesion hydrogels for monitoring and diagnosis of oral and gastrointestinal diseases. A. Envisaged application of the leak-detecting sealant hydrogel patches enabling non-invasive postoperative surveillance of anastomoses using port-able, pocket handheld ultrasound probes. Reproduced by permission from [116], copyright 2022, nature portfolio. B. The dual modality gastric leak-detecting smart sealant allows unambiguous early leak detection via the two clinically most favorable imaging modalities, computed tomography and ultrasound. Reproduced by permission from [117], copyright 2023, BMC. C. Design and mechanism of the magnetic living hydrogels localized and retained in the intestine. Reproduced by permission from [118], copyright 2021, Wiley.

Alexandre H. C. Anthis et al. introduce a comprehensive solution in the guise of a modular, intelligent suture-support sealant patch. This patch is engineered to both contain and detect gastrointestinal leaks at an early stage. The sensing elements within the patch, which are responsive to pH and/or enzymes and thus triggerable, can be non-invasively probed via point-of-need ultrasound imaging [116]. The hydrogel patch is fabricated in a layer-by-layer manner. It is composed of a tissue-contact layer that imparts wet-adhesion functionality, a functional layer, and an outer non-adhesive backing layer. When gastric contents leak out, they interact with the hydrogel, triggering the formation of a substantial number of bubbles. This, in turn, generates an easily distinguishable ultrasound signal, enabling the monitoring of anastomotic leakage within the gastrointestinal tract (Figure 10A). Benjamin Suter et al. introduce a gastric-fluid-responsive, dual-modality, electronic-free leak-sensor system that can be integrated into surgical adhesive suture-support materials. The leak sensors consist of high-atomic-number carbonates embedded within a polyacrylamide matrix. When these sensors are exposed to gastric fluid, the carbonates are converted into gaseous carbon dioxide (CO₂). The CO₂ bubbles become entrapped within the hydrogel matrix. This entrapment causes a distinct increase in echogenic contrast, which can be detected by a low-cost and portable ultrasound transducer. Simultaneously, the dissolution of the carbonate species and the subsequent diffusion of the cation result in a significantly reduced contrast in computed tomography (CT) imaging. This innovative leak-sensor system offers a novel approach for detecting leaks during surgical procedures, leveraging the unique responses to gastric fluid to provide clear signals across two different imaging modalities without the need for complex electronics (Figure 10B) [117]. Xinyue Liu et al. have developed an ingestible magnetic hydrogel carrier with the aim of localizing synthetic microbes and safely prolonging their residence within the gastrointestinal (GI) tract. This serves two key functions: facilitating health monitoring and enabling sustained drug release. The carrier is designed to transport diagnostic microbes to specific intestinal locations [118]. The magnetized metal particles are incorporated into the hydrogel matrix, and the fixed-point wet adhesion of the hydrogel is realized through wearable magnets. This design successfully enables long-term and convenient monitoring of intestinal bleeding (Figure 10C). S. Collaud et al. engineered a bioadhesive hydrogel loaded with hexylaminolevulinate (HAL) for targeting the esophageal lining [119]. To render the mucoadhesive properties of the formulations visible, a blue dye was incorporated as a contrast agent. This approach allows for the sensitive visualization of intestinal metaplasia, dysplasia, and early carcinoma in Barrett’s esophagus.

Innovative mucoadhesive hydrogels are revolutionizing diagnostics in gastrointestinal medicine with their multifunctional capabilities. For example, gastric-fluid-responsive hydrogels containing high-atomic-number carbonates generate CO₂ upon exposure to fluid. This CO₂ production enhances ultrasound contrast for leak detection, while concurrently modulating computed tomography (CT) imaging. Similarly, bioadhesive hydrogels loaded with contrast agents like hexylaminolevulinate (HAL) enable the precise visualization of early neoplastic changes in Barrett’s esophagus. These advancements highlight the transformative potential of such materials in non-invasive disease detection and monitoring, presenting new and effective ways to diagnose and manage gastrointestinal disorders.

### 4.2. Treatment of mucosal defect of the oral cavity and gastrointestinal tract

Mucosal defects within the oral and gastrointestinal tracts pose substantial clinical challenges. These defects stem from various factors, including trauma, surgical procedures, or inflammatory conditions. In response, advanced hydrogel-based materials are emerging as highly versatile solutions. These materials can be customized to possess specific properties, enabling effective wound adhesion, protection, and the acceleration of healing processes within the dynamic and harsh environments characteristic of the oral and gastrointestinal mucosa.

Oral mucosal defects are the preeminent among oral mucosal diseases. More than 30% of adults, and an even greater proportion of children, experience oral mucosal defects induced by dentofacial surgery, immunological factors, or trauma [120]. Kaize Su et al. engineered a novel hydrogel which demonstrates outstanding wet-tissue adhesion performance in an in-vivo setting (using the rat tongue model). This hydrogel offers a promising and comprehensive therapeutic alternative, holding significant potential for the clinical management of oral mucosal defects [121]. Jiwei Sun et al. developed a mussel-inspired multifunctional hydrogel adhesive [122]. This material integrates pH-responsive noncovalent anchoring to wet tissues, enhanced mechanical strength, improved structural conformity, and biomimetic antioxidase activity, thereby meeting the specific requirements for effective protection and healing of oral wounds under diabetic conditions.

**Figure 11.**
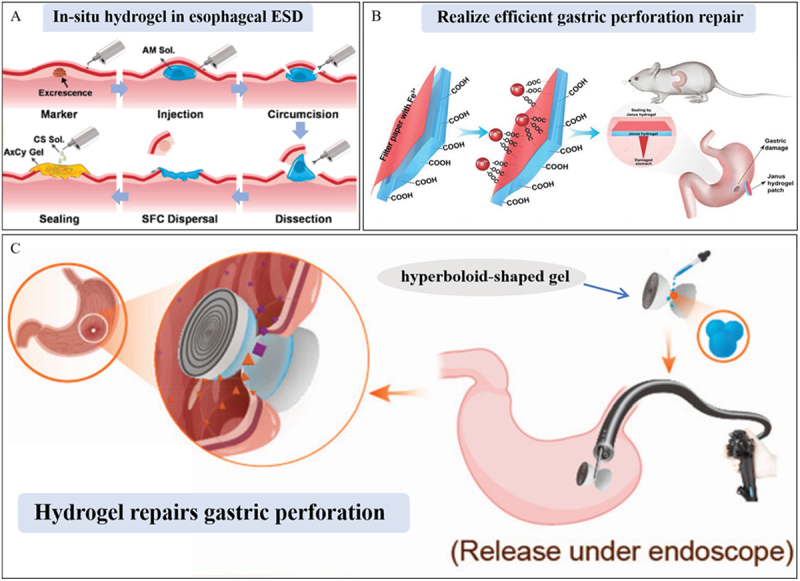
Application of wet adhesion hydrogels in mucosal defects. A. Schematic diagram showing the design principle of multifunctional hydrogel and its application in canine esophageal ESD. Reproduced by permission from [123], copyright 2023, Elsevier. B. The Janus hydrogel can realize efficient gastric perforation repair on mice. Reproduced by permission from [124], copyright 2022, Elsevier. C. The compressible hyperboloid-shaped gel packs gastric perforation and promotes tissue healing [125]. Reproduced by permission from [125], copyright 2022, American Chemical Society.

For endoscopic submucosal dissection (ESD) of gastrointestinal tumors and premalignant lesions, a submucosal fluid cushion is essential to achieve mucosal uplift prior to dissection. Moreover, effective wound care, involving wound closure and prompt healing after the operation, is crucial. Xiong-Xin Lei et al. report on a two-component in-situ hydrogel synthesized from maleimide-based oxidized sodium alginate and sulfhydryl carboxymethyl-chitosan. Sodium alginate (SA) and carboxymethyl chitosan (CMCS) are modified into two derivatives, maleimide oxidized sodium alginate ADA-Mal (abbr. to AM) and mercaptocarboxymethyl chitosan CMCS-SH (abbr. to CS). The canine esophageal ESD model verified that this in-situ hydrogel furnished excellent mucosal uplift and wound-closure effects. Additionally, it significantly expedited wound healing following ESD by promoting re-epithelization and extracellular matrix (ECM) remodeling (Figure 11A) [123]. Kazuhiro Nagasaka et al. developed a injectable Cat-PBA-ApGltn hydrogels, which are based on catechol group-modified Alaska pollock gelatin (Cat-ApGltn) and phenylboronic acid-modified Alaska pollock gelatin (PBA-ApGltn) [126]. This hydrogel exhibits a good mucosal elevation effect and has broad application prospects in ESD. Jing Yu et al. report the synthesis of a potent hydrogel adhesive through the free-radical polymerization of N-acryloyl aspartic acid (AASP) in a facile and straightforward manner (Figure 11B) [124]. They engineered this hydrogel to adhere robustly to the wound surface, functioning as a robust physical barrier for hemostasis. Additionally, they applied a paper-based Fe^3 +^ transfer-printing method to fabricate a PAASP-based Janus hydrogel patch featuring both adhesive and non-adhesive surfaces. In a murine gastric-perforation model, this patch enabled the concurrent achievement of wound healing and postoperative anti-adhesion. Shuang Liu et al. designed acid-resistant tough and elastic double network hydrogel, this hydrogel has good wet adhesion, can withstand the mechanical movement of the stomach wall, and can adjust the pH of the wound site, making it more suitable for mucosal healing (Figure 11C) [125]. Sitong Lu et al. developed a gastric-acid-responsive hydrogel, CS-NAC/alginate/tilapia collagen peptide (CS-NAC/ALG/TCP). In mouse stomachs, this hydrogel, CS-NAC/ALG/TCP, effectively improved the oxidative-stress status of the gastric mucosa. It down-regulated the expression of inflammatory factors and enhanced the production of gastric protective factors such as prostaglandin E2 (PGE2) and nitric oxide (NO). As a result, it mitigated alcohol-induced inflammation and safeguarded against gastric mucosal injury [127].

The evolution of multifunctional hydrogels has revolutionized the management of oral and gastrointestinal mucosal defects. In-situ hydrogels, for instance, have emerged as powerful tools for achieving efficient mucosal uplift and promoting wound healing following endoscopic submucosal dissection. Acid-resistant hydrogels, on the other hand, are designed to endure mechanical stress and regulate pH levels, thereby facilitating enhanced gastric healing. Notably, innovative constructs like Janus hydrogel patches represent a significant leap forward. These patches are engineered to perform dual functions: providing wound adhesion while simultaneously preventing post-operative adhesions. Gastric acid-responsive hydrogels, meanwhile, actively intervene in the inflammatory and oxidative-stress processes within the gastric environment. Collectively, these advancements vividly demonstrate the transformative potential of hydrogels in mucosal repair and regeneration. They lay a solid foundation for improving clinical outcomes, offering new hope for patients suffering from mucosal-related pathologies.

### 4.3. The treatment of oral and gastrointestinal tumors

Gastrointestinal cancer is one of the most malignant tumors with high morbidity and mortality, especially colorectal cancer, which has become the second leading cause of cancer-related deaths worldwide [128]. Oral cancer is one of the most common cancers in the world, with more than 300,000 diagnosed cases each year, a 5-year survival rate of only 40–60%, and a poor prognosis. Exploring new strategies for early diagnosis and treatment of oral cancer is key to improving survival rates [129]. Conventional chemotherapy and surgical procedures are often accompanied by a plethora of complications. In contrast, targeted drug therapy emerges as an alternative approach. It has the potential to mitigate the toxicity associated with systemic chemotherapy. Moreover, this form of therapy enables the sustained release of chemotherapy drugs precisely at the site of the target tumor, thereby enhancing therapeutic efficacy while minimizing adverse effects on the body as a whole [130]. Wet-adhesion hydrogels possess the ability to undergo spontaneous degradation. This characteristic obviates the need for surgical excision. Additionally, these hydrogels ensure the long-term and regional release of the encapsulated cargo, thereby maintaining a high drug concentration at the desired site [131]. In the field of precision treatment of gastrointestinal tumors, wet adhesion hydrogels have broad clinical application prospects [132,133].

Di-Wei Zheng et al. found that antitumor responses were enhanced by the subcutaneous delivery of an adhesive hydrogel incorporating silver nanoparticles [134]. Their findings indicate that wet-adhesive hydrogels can be engineered to regulate the human microbiota, thereby enhancing antitumor immune responses. This hypothesis has been experimentally validated in mice models of oral squamous cell carcinoma. Lan Chen et al. developed implantable in situ vaccine hydrogel (APHP-CCCA) for the prevention of postoperative recurrence of oral squamous cell carcinoma (OSCC) [135]. This hydrogel system stimulates macrophages to achieve a systemic anti-tumor immune response. Xing Wang et al. engineered a dry polyacrylic acid (PAA)-chitosan (CHI)-5-aminolevulinic acid (ALA) interpenetrating polymer network hydrogel patch (designated PACA) with robust adhesion [136]. This PACA patch achieves rapid, stable adhesion to wet mucosal surfaces and enables targeted drug delivery to prevent the progression of potentially malignant oral lesions.

Wen Li et al. fabricated a bacterial hydrogel by incorporating Thiobacillus denitrificans into the hydrogel, enabling targeted adhesion to the colon. The bacteria loaded within the HA-SH hydrogel are capable of scavenging excessive hydrogen sulfide (H₂S) in colon cancer. This, in turn, promotes the normalization of tumor vasculature, ultimately inhibiting tumor progression [137]. Ke Gong et al. engineered a thermosensitive hydrogel that is formulated from F127 and tannic acid, functioning as an effective carrier for loading nanoparticles (NPs). Once the hydrogel makes contact with esophageal tissue, it experiences a phase transformation, evolving into a gelatinous consistency. This change enables it to adhere robustly to the esophageal lining. As a result, it can facilitate the prolonged and sustained release of the encapsulated therapeutic agents. By doing so, it effectively contributes to the treatment of esophageal cancer, exerting a targeted and long-lasting therapeutic impact on the affected area [138]. Wen Chen designed a 5-fluorouracil and cis-platinum co-delivery system based on a biodegradable temperature-sensitive hydrogel for intraoperative adjuvant combination chemotherapy of gastric cancer [139]. Neoantigen-based cancer vaccines show great potential in cancer immunotherapy due to their ability to induce effective and long-lasting anti-tumor immunity [140–143]. Biomaterials, notably hydrogels, have become prominent in tumor immunotherapy due to their excellent biocompatibility and customizable features. Hydrogels’ unique structures can be precisely tailored, and their biocompatibility enables seamless integration into the tumor microenvironment. This makes them ideal for delivering immunomodulatory agents, thus offering new strategies in cancer treatment [144–146]. Ying Yang et al. grafted tumor cell lysate onto polydopamine nanoparticles as nano-vaccine (TCLN) and fabricated alginate hydrogel loaded with Endostar (EH), this hydrogel is effective in improving the anti-tumor immune response against colon tumors [147].

In clinical practice, surgical resection is the primary treatment for most solid tumors. The status of surgical margins, specifically the presence of residual tumor tissue, often dictates tumor-related survival rates and the likelihood of recurrence. As such, accurately defining tumor margins is crucial for achieving complete resection. Seon Sook Lee et al. propose the application of an indocyanine green-loaded alginate hydrogel system as a fluorescence surgical marker for precise laparoscopic operations [148]. The design of this hydrogel facilitates the precise resection of gastrointestinal tumors.

**Figure 12.**
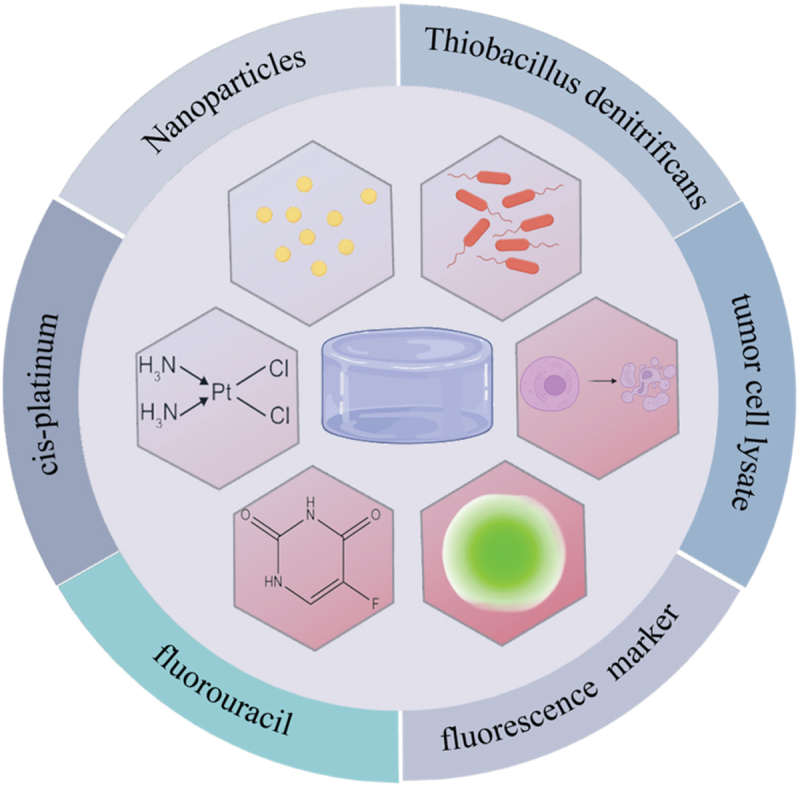
Ingredients encapsulated in hydrogels for the treatment of oral and gastrointestinal tumors.

Emerging mucoadhesive hydrogels are revolutionizing oral and gastrointestinal tumor treatment strategies. Their distinct advantages lie in localized drug delivery, immune modulation, and targeted interaction with the tumor microenvironment (Figure 12). For example, hydrogels loaded with silver nanoparticles boost antitumor immunity in oral cancer, and bacterial hydrogels scavenge excess H₂S to normalize blood vessels in colon cancer, highlighting their remarkable versatility. Thermosensitive hydrogels ensure the sustained release of therapeutics. Multifunctional platforms that combine chemotherapeutics, nano-vaccines, or fluorescent markers are propelling precision oncology forward. Altogether, these advancements emphasize the pivotal and transformative role of hydrogels in enhancing the effectiveness and precision of cancer therapy.

### 4.4. Inflammatory diseases of the oral and gastrointestinal diseases

In recent years, people have paid increasing attention to inflammatory diseases of the digestive system. On the one hand, chronic inflammation is an important factor in tumor development. On the other hand, digestive system inflammatory disease easily recurs and is not easily cured, seriously affecting quality of life [149]. The therapeutic potential of current anti-inflammatory drugs can be maximized by precisely controlling their activity both spatially and temporally. This approach holds great promise as a solution for treating inflammatory disorders of the digestive tract.

**Figure 13.**
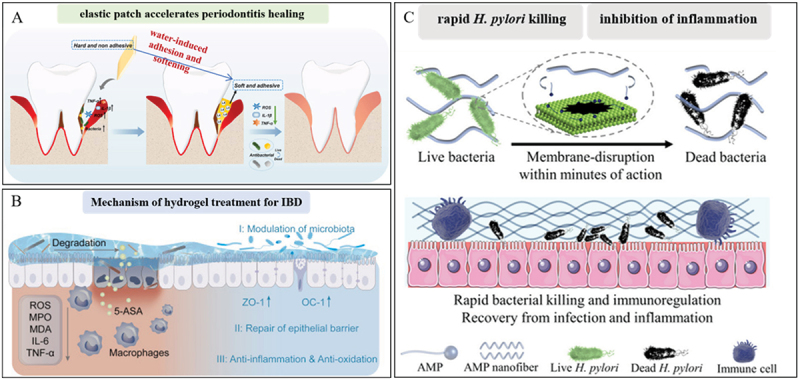
The role of hydrogels in oral and gastrointestinal inflammatory diseases. A. Illustration of the proposed mechanism of PolyLA-GelMA patch for promoting periodontitis healing. Reproduced by permission from [150], copyright 2024, Elsevier. B. Inulin gel composite for IBD treatment by restoring intestinal homeostasis. Reproduced by permission from [151], copyright 2024, Elsevier. C. The C12G2 hydrogel can regulate immune responses, leading to improved therapeutic efficacy. Reproduced by permission from [152], copyright 2024, American Chemical Society.

Ying Qi et al. report a wet-responsive methacrylated gelatin-stabilized co-enzyme polymer poly (α-lipoic acid) (PolyLA)-based elastic patch with water-induced adhesion and softening features (Figure 13A) [150]. The hydrogel allows durable adhesion to oral periodontal tissue and continuous release of LA-based bioactive small molecules in periodontitis wounds without resorting to external drugs. Xiao Chen et al. have developed a new injectable cationic hydrogel (OP). The hydrogel successfully mitigated periodontal bone loss in mice by capturing pathogenic molecules such as lipopolysaccharides and free DNA and blocking inflammation mediated by the TLR4/9 pathway [153]. Dong-Heon Ha et al. developed an esophagus-derived decellularized extracellular matrix hydrogel and it has been shown to have a good therapeutic effect in a rat model of radiation esophagitis [154]. Hydrogels with good shear thinning properties are of great interest in this area [155]. This hydrogel has good flowing property and a certain viscosity so that it can be 3D printed into a suitable shape and effectively adhere to the esophageal surface. Xiayi Xu et al report the development of a hydrogel based on thiourea-catechol reaction to enhance the bio-adhesion. They further report the robust adhesion of our hydrogels to acidic gastric tissues and effectively inhibit stomach inflammation [156]. Antimicrobial peptides (AMPs) have been reported to possess unique advantages against antimicrobial-resistant bacteria due to their rapid physical membrane disruptions and anti-inflammation/immunoregulation properties [157]. Zhuangzhuang Zhang et al. developed a combination of olsalazine-based nanoneedles and microbiota-regulating inulin gel to reshape intestinal homeostasis and relieve inflammation. The hydrogel displayed a macroporous structure, improved bio-adhesion, and enhanced colon retention after oral administration (Figure 13B) [151]. Haoning Gong developed an AMP hydrogel, which can be orally administered for the treatment of Helicobacter pylori (*H. pylori*) infection suppresses the expression of pro-inflammatory cytokines, and uniquely promotes inflammation resolution (Figure 13C) [152]. IBD (chronic intestinal inflammatory disease) in digestive system diseases such as ulcerative colitis (a chronic, multifactorial, and inflammatory disease of the colon) and Crohn’s disease [158]. Xinyue Ge et al. report a multifunctional mechanically-resilient self-healing hydrogel for effective ulcerative colitis treatment, DA moieties in hydrogel allow firm adhesion of the hydrogel to the ulcerative lesions after in-situ implantation [94]. Crohn’s disease (CD) is a complex, multifactorial, immune-mediated illness, and perianal fistulas (PAF) represent a severe complication of Crohn’s disease [159]. Ling Li et al. report an injectable, biodegradable, mechanically fragmented nanofiber-hydrogel composite (mfNHC) loaded with adipose-derived stem cells (ADSCs) for the treatment of fistulas in a rat model of CD-PAF [160]. The homeostatic balance between gut microbiota and the mucosal immune system is severely disrupted in the context of UC, eventually leading to sustained mucosal damage [161,162]. Meanwhile, there is concrete evidence that the enteric microbiome in UC patients is usually in an aberrant state with overwhelming harmful flora, which may cross the damaged intestinal mucosal barrier to aggravate local inflammation [163,164]. Hong Wen et al. developed a multifunctional biomimetic hydrogel as artificial colonic mucosa, which can readily establish a bioadhesive barrier at the ulcer site and restore local immune and microbial homeostasis to exert potent anti-inflammatory effect [165].

Mucoadhesive hydrogels are revolutionizing therapeutic approaches for oral and gastrointestinal inflammatory diseases. Their distinctive capacity to enable localized and sustained treatment is key. For instance, bioactive molecule-releasing patches for periodontitis and hydrogels engineered to adhere strongly and exert anti-inflammatory effects in gastric and intestinal tissues are innovative solutions. These address crucial hurdles in managing chronic inflammation. Advanced hydrogel designs, such as those that regulate the microbiota and multifunctional self-healing materials, play a dual role. They not only facilitate mucosal repair but also re-establish microbial and immune balance. Altogether, these mucoadhesive hydrogels signify a paradigm shift. They present precision therapies for complex inflammatory disorders, including inflammatory bowel disease (IBD), radiation esophagitis, and Crohn’s disease, heralding a new era in the treatment of such conditions.

### 4.5. Wet adhesion hydrogel ameliorates oral and gastrointestinal dysbiosis

Periodontitis is an inflammatory disease caused by an imbalance in the periodontal microbial ecosystem. Traditional treatment methods not only kill pathogenic bacteria but also inhibit the growth of beneficial bacteria, thereby disrupting the balance of the oral microbial ecosystem [166]. Mucus, a viscoelastic fluid, coats and safeguards the gastrointestinal (GI) tract. The mucus secreted by the cells within the digestive tract is of utmost importance for maintaining the microbiome homeostasis in this region [167]. A healthy gut microbiota plays a pivotal role in promoting robust intestinal immunity. Consequently, emerging biomaterials that aid in preserving the balance of the microbiota have emerged as a focal point of research. Wet-adhesive hydrogels, in particular, can function as a cutting-edge delivery platform. By integrating with the immunomodulatory signals of symbiotic microorganisms, they offer a more comprehensive strategy to address a variety of issues stemming from microbiota dysbiosis [168].

**Figure 14.**
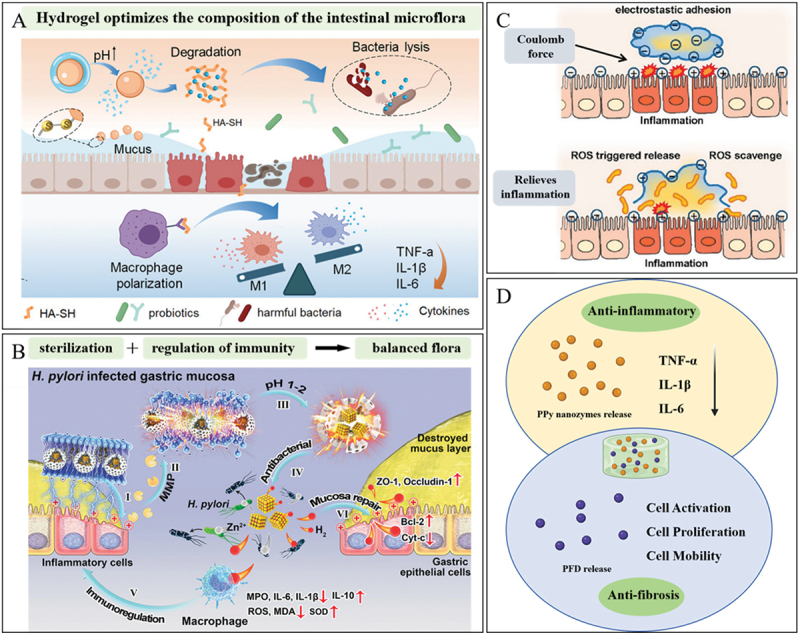
Wet adhesion hydrogel ameliorate intestinal dysbiosis. A. Oral administrated HA-SH-Ag/Alginate-ca microspheres (HAMs) target colon to collapse and release HMs. Reproduced by permission from [170], copyright 2021, Wiley. B. Schematic diagram of inflammation-targeted hydrogel sterilization. Reproduced by permission from [171], copyright 2021, Wiley. C. Negatively charged HA-LR hydrogel preferentially adheres to the positively charged inflamed epithelium, then releases bacteria locally triggered by ROS, thus to alleviate inflammation. Reproduced by permission from [172], copyright 2022, Elsevier. D. The PPy/PFD@Inulin gel exerts multifaceted regulation on the mechanisms associated with IBD and intestinal fibrosis.

Yi Wang et al. designed a kind of nanoparticle of PSCLR (living Lactobacillus rhamnosus (LR) modified with chitosan (CS), hyaluronic acid (HA), and puerarin) [169]. The live probiotic nanoparticles are subsequently encapsulated within hydrogel microspheres to create PSCLR-HA Gel toothpaste with flora regulation and immune recovery properties. Hua Liu et al. designed a colon‐targeted adhesive core-shell hydrogel microsphere fabricated by the ingenious combination of advanced gas‐shearing technology and ionic diffusion method, which can congregate on colon tissue regulating the gut immune‐microbiota microenvironment in IBD treatment (Figure 14A) [170]. Helicobacter pylori (H. pylori) infection is the leading cause of chronic gastritis, peptic ulcer, and gastric cancer [173,174]. A pH-responsive metal-organic framework hydrogen-generation nanoparticle (Pd (H) @ ZIF-8) is reported, which is encapsulated with ascorbate palmitate (AP) hydrogel (Figure 14B) [171]. The outer AP hydrogel can target and adhere to the inflammatory site and released Pd(H) @ ZIF-8 nanoparticles are further decomposed by gastric acid to generate zinc ions (Zn2+) and hydrogen, thus effectively killing H. pylori, avoiding causing severe bacterial resistance and intestinal flora loss. Some strains present in the human gut are known as ‘probiotics’ and can be consumed alive to promote health benefits and to keep the intestinal mucosal layer safe from pathogens, thereby boosting the immune system [175,176]. Precise delivery and on-demand release of probiotics are particularly important to improve intestinal dysbiosis. To address this problem, a ROS-responsive hydrogel based on hyaluronic acid (HA) was developed for encapsulation and targeted delivery of probiotics (Figure 14C) [172]. It preferentially adheres to the site of inflammation, and excess reactive oxygen species (ROS) produced by inflamed colon tissue selectively cleaves thioaldehyde bonds, leading to hydrogel degradation and local probiotic release. Xiangjing Cao and colleagues developed a probiotic inulin hydrogel incorporating polypyrrole (PPy) nanozyme and the antifibrotic drug pirfenidone (PFD), namely the PPy/PFD@Inulin gel (Figure 14D) [177]. This gel was ingeniously designed to concurrently improve inflammatory bowel disease (IBD) and its associated fibrotic complications. Once orally administered, the inulin-based gel matrix effectively extends the duration of polypyrrole nanozymes and pirfenidone within the gastrointestinal tract. It attains the therapeutic objectives by continuously scavenging reactive oxygen and nitrogen species (RONS) and modulating the intestinal microbiota.

Mucoadhesive hydrogels are emerging as transformative tools in the management of inflammation-driven dysbiosis in the oral and gastrointestinal tract. From colon-targeted core-shell hydrogels modulating the immune-microbiota axis in IBD to pH-responsive hydrogels releasing zinc ions and hydrogen for H. pylori eradication, these innovations highlight the precision and adaptability of hydrogel systems. Furthermore, ROS-responsive hydrogels enable targeted delivery of probiotics, promoting microbial homeostasis and immune resilience. In summary, by simultaneously addressing the intertwined challenges of dysbiosis and inflammation, these advanced mucoadhesive hydrogels present a highly promising approach. They offer the potential to restore gut health and effectively mitigate the progression of diseases associated with gastrointestinal dysregulation.

## 5. Challenges and future perspectives

### 5.1. Key challenges

**Figure 15.**
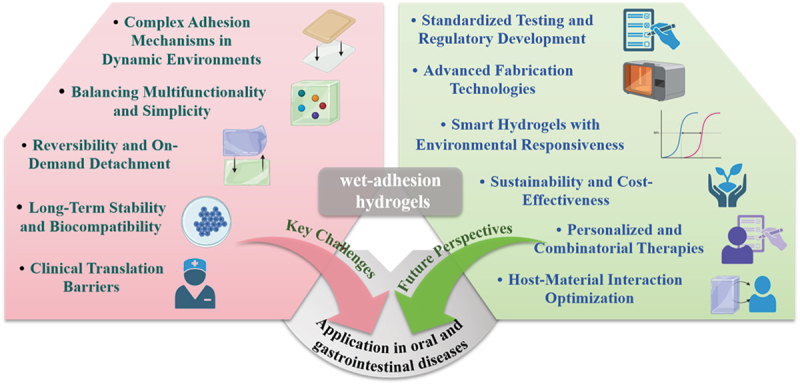
Key challenges and future perspectives in wet-adhesion hydrogels.

While wet-adhesive hydrogels hold transformative potential for oral and gastrointestinal applications, their clinical translation is hindered by multifaceted challenges. Current strategies – such as catecholamine-based adhesion, hydrophobic modifications, and microneedle designs – have advanced interfacial bonding, yet the dynamic interplay of hydrogen bonding, electrostatic forces, and mechanical interlocking in the harsh, ever-shifting gastrointestinal milieu remains elusive. For instance, catechol groups, though biomimetic, are vulnerable to oxidation at physiological pH, undermining adhesion stability. Simultaneously, hydrogels must balance long-term structural integrity against gastric acid, enzymatic activity, and peristalsis, where uncontrolled swelling or premature degradation risks functional compromise, while synthetic polymer byproducts demand rigorous biocompatibility evaluation. The integration of multifunctional properties (e.g. antibacterial, antioxidant) introduces further complexity: excessive functionalization risks manufacturing bottlenecks, cost escalation, and systemic toxicity, as seen with nanoparticle accumulation. Translational barriers persist as small animal models inadequately replicate human gastrointestinal anatomy and microbiota, while scaling production without sacrificing consistency in adhesion strength or drug release kinetics remains daunting. Regulatory frameworks, too, lag in addressing dynamic mucosal environments. Finally, while robust adhesion is critical, the absence of reversible, on-demand detachment mechanisms limits applications requiring temporary interfaces, such as biosensors or wound dressings. Addressing these challenges demands interdisciplinary innovation to harmonize material science with biological complexity, ensuring hydrogels evolve from promising prototypes into reliable clinical solutions (Figure 15).

### 5.2. Future perspectives

The next generation of wet-adhesive hydrogels promises to revolutionize gastrointestinal therapeutics through innovations that harmonize environmental responsiveness, precision engineering, and biological integration. Stimuli-responsive systems, engineered to adapt to pH, temperature, or reactive oxygen species (ROS), could enable site-specific drug release and adhesion – exemplified by Prussian blue nanoparticles (PBNPs) or tannic acid (TA) scaffolds that exploit oxidative stress in inflamed tissues to trigger therapeutic effects while sparing healthy mucosa. Advances in fabrication, such as 4D-printed biomimetic architectures inspired by octopus tentacles or barnacle amyloid nanostructures, may refine hydrogel topology and adhesion dynamics, while microfluidic and nanotechnological approaches could orchestrate spatially controlled drug delivery or real-time diagnostic feedback. Beyond structural ingenuity, future designs must prioritize dynamic host-material crosstalk, modulating mucosal microenvironments to restore microbiome equilibrium via probiotic encapsulation or recruiting endogenous stem cells for regenerative repair, synergized by immunomodulatory cues to combat chronic pathologies like ulcerative colitis. Personalized therapies could emerge from hydrogels tailored with patient-derived cells or tumor neoantigens, transforming cancer immunotherapy through localized immune activation. However, clinical translation demands rigorous standardization of adhesion metrics and biocompatibility under physiomimetic conditions, necessitating interdisciplinary collaboration to bridge laboratory innovation and clinical practice. Finally, sustainability must underpin this evolution, with eco-friendly hydrogels derived from renewable biopolymers (e.g. lignin, chitosan) and scalable, solvent-free syntheses ensuring accessibility without ecological compromise. Together, these converging advances herald an era where hydrogels transcend static biomaterials, evolving into intelligent, adaptive systems that seamlessly interface with biology to diagnose, treat, and heal with unprecedented precision (Figure 15).

## 6. Conclusion

Wet-adhesive hydrogels represent a paradigm shift in treating oral and gastrointestinal diseases, offering unparalleled adaptability to dynamic mucosal environments. However, overcoming challenges in adhesion stability, biocompatibility, and clinical translation demands interdisciplinary innovation. By embracing smart designs, advanced manufacturing, and a deeper understanding of host-material interactions, next-generation hydrogels could achieve precision therapy, real-time diagnostics, and microbiome engineering – ushering in a new era of gastrointestinal medicine. Collaborative efforts across academia, industry, and healthcare systems will be pivotal in transforming these biomaterials from laboratory curiosities into life-saving clinical tools.
